# Brain Activity Elicited by Positive and Negative Feedback in Preschool-Aged Children

**DOI:** 10.1371/journal.pone.0018774

**Published:** 2011-04-19

**Authors:** Xiaoqin Mai, Twila Tardif, Stacey N. Doan, Chao Liu, William J. Gehring, Yue-Jia Luo

**Affiliations:** 1 Center for Human Growth and Development, University of Michigan, Ann Arbor, Michigan, United States of America; 2 Department of Psychology, University of Michigan, Ann Arbor, Michigan, United States of America; 3 Department of Psychology, Boston University, Boston, Massachusetts, United States of America; 4 State Key Laboratory of Cognitive Neuroscience and Learning, Beijing Normal University, Beijing, China; University College London, United Kingdom

## Abstract

To investigate the processing of positive vs. negative feedback in children aged 4–5 years, we devised a prize-guessing game that is analogous to gambling tasks used to measure feedback-related brain responses in adult studies. Unlike adult studies, the feedback-related negativity (FRN) elicited by positive feedback was as large as that elicited by negative feedback, suggesting that the neural system underlying the FRN may not process feedback valence in early childhood. In addition, positive feedback, compared with negative feedback, evoked a larger P1 over the occipital scalp area and a larger positive slow wave (PSW) over the right central-parietal scalp area. We believe that the PSW is related to emotional arousal and the intensive focus on positive feedback that is present in the preschool and early school years has adaptive significance for both cognitive and emotional development during this period.

## Introduction

Children's ability to regulate their cognitive performance and emotional expressions undergoes dramatic improvements in the preschool and early school years. Part of this change requires children to learn from external feedback, yet a challenge for parents and educators alike is the difficulty of guiding children's learning through a focus on correcting children's mistakes. Not only do preschoolers tend to perseverate in their errors, they react with a variety of negative emotions to explicit corrections and still have trouble identifying, preventing, and correcting the mistakes [1]. Moreover, as evidenced by a wide variety of educational approaches, an emphasis on increasing children's awareness of the positive or “correct” modeling of the desired behaviors often leads to both improved affect and motivation for learning, and improved performance as well [2], [3].

An important step in developing a theoretical account of this difference in children's ability to incorporate positive vs. negative feedback is to identify the neural processes that give rise to the behavioral phenomena. Here, we report a study of event-related brain potentials (ERPs) elicited by feedback processing in preschoolers. Our goal was to use a task analogous to those used to study feedback processing in adults to determine (a) whether the feedback-related ERP effects seen in adults would also be evident in children and (b) whether those effects would show a greater sensitivity to positive feedback than to negative feedback.

In adults, many studies have investigated feedback processing during gambling tasks using the ERP [4], [5]. For instance, in Gehring and Willoughby's study [4], participants were asked to choose one of two squares containing either the numeral 5 or 25, and were then shown the outcome of winning or losing the amount of money indicated by the chosen numeral. Results showed a medial frontal negativity (MFN), which is now usually called the feedback-related negativity (FRN), peaking at about 270 ms. The FRN is larger after negative outcomes (monetary losses) than after positive outcomes (monetary wins). It appears to be generated by the anterior cingulate cortex (ACC), and may reflect a negative reinforcement learning signal conveyed to the ACC via the mesencephalic dopamine system, which is used by the ACC to modify behavior [6].

Few studies have systematically examined FRN and its development in children, and results have been mixed. Eppinger, Mock, and Kray [7] reported a larger FRN for negative feedback in children aged 10–12 years compared to adults in a probability learning task, whereas no age differences were found for positive feedback. The authors interpreted these results as children being more sensitive to negative feedback during learning. However, Groen et al. [8] did not observe this in children of a similar age. They interpreted the absence of FRN as related to the possibility that the feedback stimuli (i.e., green and red squares) used in their study were not motivationally salient enough for the children. Nevertheless, it is also possible that the absence of FRN in children is due to the late maturation of the ACC. This has been evidenced in non-human primate studies [9], [10], a functional Magnetic Resonance Imaging (fMRI) study [11] and some developmental ERP studies on error-related negativity, which may also be generated by the ACC [12], [13], [14].

In addition to the FRN, a long-latency positivity has been reported in studies of children's ERPs to feedback and is thought to reflect affective processing. van Meel et al. [15] elicited ERPs for positive and negative feedback using a guessing game and found a long latency ERP component at 450–500 ms, which was more positive for losses than gains in children aged 8–12 years. They proposed that the late positivity might be related to emotional processing. Similarly, Groen et al. [16] also reported a late positivity at 450–1000 ms, with a central-parietal maximum, which was larger for negative feedback than positive feedback in children aged 10–12 years. Previous adult ERP studies have not observed these long latency differences between negative and positive feedback.

In the present study, we designed a prize-guessing game which was analogous to adult “gambling” tasks but more child-friendly, because children have neither clear conceptions of money nor of the relative quantities used in adult tasks. To inspire children's interest in the stimuli, we adapted the gambling task to make it more similar to “prize” tasks used to measure emotion regulation responses in behavioral studies of preschoolers [17], [18]. In this task, participants were initially asked to sort a set of prizes and to rank-order them in decreasing order of preference (e.g., from attractive tambourines to bottle caps). Cole [17] in her study reported that in a debriefing interview all children acknowledged positive feelings about receiving the first-ranked prize and the majority of the children (80%) acknowledged negative feelings (sad/mad/yukky) about the last-ranked one. Therefore, we would expect such a task to elicit differential feelings about each type of prize in the present study, regardless of the fact that they were both prizes. Specifically, we expected to observe long latency ERP components related to emotional processing in preschool-aged children using this child-friendly prize-guessing game. According to the study of Eppinger et al [7], we might also observe an FRN response if it is indeed observable in preschoolers since we used more motivating stimuli. However, it is also possible that we might not be able to observe a larger FRN for negative feedback in such young children because of the relatively late maturation of the ACC.

## Methods

### Participants

Eighteen healthy children aged 4–5 years (mean age 53.95±4.21 months; 9 females) participated in the study. Two children did not finish the experiment (one due to equipment problems, another who could not sit still). In addition, the data from three other children were not complete because of technical errors or excessive data artifacts. Therefore, 16 children with behavioral data and 13 (mean age 53.20±4.08 months; 5 females) with ERP data were used in the final analyses. There were no significant age differences between the 13 included and 5 excluded children for ERP analysis (*t*(16)  = 1.24, *p* = 0.23). Children were enrolled in the study with written consent of their parents and were paid for their participation. Oral assent was also obtained from all children. The study was approved by the Institutional Review Boards at the University of Michigan and Beijing Normal University.

### Task and procedures

Each child was shown ten potential prizes, which included “good” and “bad” prizes, and was asked to rank-order them by picking the best, second best, and so on until all ten were ranked. Then the experimenter chose the first three as good prizes and the last three as bad prizes, and the child was asked to play the prize-guessing game with those prizes as feedback.

#### Prize-Guessing Game (Figure 1A)

Each child was shown two boxes on a computer screen and was told that one of them contained a good prize and the other a bad prize. The color of the choice box could be red, green, blue or yellow. The child was then asked to guess which box the good prize was in, with no apparent links between the color of the choice and the prize (“good” vs. “bad”) boxes. Once the child made a choice, the experimenter recorded it by pressing a button which then displayed the box the child chose, and then a second button to show the prize. Following a 1000 ms interval, the prize was revealed and remained on the screen for 2000 ms. The child was told that if he or she guessed correctly, s/he would get a red star; if incorrectly, a black star. That is, s/he would get a red star if s/he picked the “good prize” and get a black star if s/he picked the “bad prize” box. At the end of the experiment, if the child had more red stars than black stars, s/he would get the three good prizes; otherwise s/he would get the three bad prizes. Importantly, the children did not see the actual stars accumulated until the very end of the experiment, at which point all children got more red stars than black stars and were ultimately given the three “good” prizes.

**Figure 1:**
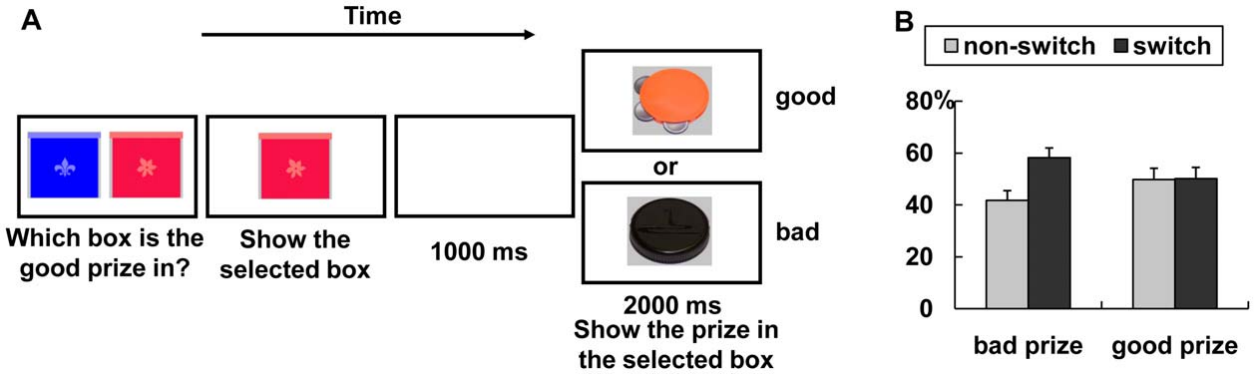
The task and behavioral responses. (A) Illustration of the prize-guessing game. (B) Behavioral responses to feedback when previous trial showed bad vs. good prizes.

The whole game involved 90 trials. Unknown to children, they received 45 “good prize” and 45 “bad prize” trials, presented in random order, regardless of which box they chose on any given trial. In addition, each child's electroencephalography (EEG) and behavior were continuously monitored across the session, so that the prize stimulus was presented only when the child was sitting still and looking at the screen. To familiarize children with the procedure and inspire their interest in participating in the formal experiment, a practice session with seven trials and another set of prizes was performed before the formal ERP experiment. The practice session was designed such that all children guessed correctly four times and incorrectly three times in randomized orders, and in the end, received good prizes for the practice session.

### ERP Recording and Analysis

EEG was recorded using a 128-channel Geodesic Sensor Net (Electrical Geodesics Inc., Eugene, OR). The EEG signal was amplified using a 0.01–70 Hz bandpass and digitized at 250 Hz. All recordings were referenced to Cz, and electrode impedances were kept below 50 kΩ. After acquisition, raw EEG was lowpass filtered below 20 Hz, and then segmented into epochs from 200 ms before to 1000 ms after the onset of the prize stimulus that was shown to the child. Trials with blink and eye movement artifacts, and trials in which more than 10 bad channels exceeding 200 µV (absolute) or 100 µV (sample to sample) were excluded. For each participant, artifact-free trials were averaged separately for good-prize and bad-prize stimuli (mean = 33.7, SD = 5.0 trials for good-prize condition and mean = 33.2, SD = 5.2 trials for bad-prize condition). The data were re-referenced against the average of all channels. The 200 ms preceding the prize stimulus served as baseline.

**Figure 2:**
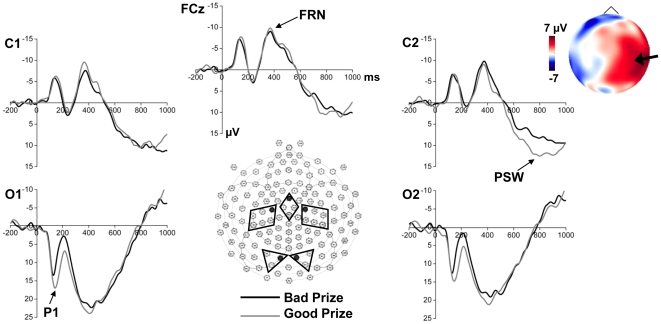
Grand-averaged ERP waveforms. The ERPs elicited by bad prizes vs. good prizes at FCz, C1, C2, O1 and O2 sensors (labels derived from the conversion chart provided by Electrical Geodesics Inc. corresponding to electrodes 6, 31, 106, 72, and 77, respectively), denoted by black dots in the map of the 128-channel geodesic sensor net. Electrode groupings used for analysis of ERP components are denoted with different shapes (a diamond for FRN, parallelograms for PSW, and triangles for P1). The topographic map on the upper right is constructed from amplitude values at 800 ms post-stimulus in difference waveforms consisting of the bad-prize waveform subtracted from the good-prize waveform. The right hemisphere differences are indicated by the arrow.

Based on previous studies [4], [7], [16] and inspection of the grand-averaged waveforms, we identified three components: FRN in middle fronto-central electrodes (6, 7, 107, and 129), P1 in occipital electrodes (66, 71, 72, 77, 84, 85), and a positive slow wave (PSW) in central parietal electrodes (31, 37, 38, 42, 43, 48, 88, 94, 99, 104, 105, 106) (Figure 2). The sets of electrode groupings for P1 and PSW were split into left and right regions for analysis. The baseline-to-peak amplitude and latency of P1 was measured in the 120–200 ms window. The mean amplitude of FRN and PSW were measured in the 350–450 ms and 650–900 ms windows, respectively. Repeated measures analysis of variance (ANOVA) using the General Linear Model procedure of SPSS, version 18 (SPSS Inc., Chicago, IL, USA) were performed on these variables. Prize (good, bad) was used as the within-subject factor for FRN, and Prize (good, bad) and Laterality (left, right) were used as the two within-subject factors for P1 and PSW. Greenhouse-Geisser corrections were applied to compensate for sphericity violations. Thirteen children constituted a relatively small sample, thus Cohen's effect sizes [19] were also calculated to ensure that the results were reliable, with Cohen's [19] suggested values of .20, .50, and .80, used to indicate small, medium, and large effect sizes, respectively.

It should be noted that the N1 was also identified in occipital electrodes (66, 71, 72, 77, 84, 85). To eliminate the effect of the preceding P1, the N1 amplitude was measured by computing the difference between the most negative peak within 200–300 ms and the preceding most positive peak within 120–200 ms after the prize stimulus. Repeated measures ANOVA showed that the N1 amplitude showed no differences between good and bad prizes. We thus do not further discuss the N1 component.

## Results

### Behavioral results

To examine whether children in the prize-guessing game applied response strategies in response to feedback, we classified each trial according to whether children choose the box on the same side as the previous trial and whether that previous trial had been a good or bad prize. Trials where children chose the side opposite to the one chosen on previous trials were labeled ‘Switch’ trials, whereas those on the same side were labeled ‘Non-switch’ trials. A 2×2 two-way repeated measures ANOVA with factors of Prize (good, bad) and Switch (switch or not) revealed an interaction effect, *F*(1,15)  = 8.31, *p* = .01. Pairwise comparisons showed that children switched their responses more frequently after being shown that their “choice” was a bad prize (58% Switch vs. 42% Non-switch, 95% confidence interval of the difference: 1%–33%, *p* = .04, Cohen's effect size = 0.55) than after a good prize (50% Switch vs. 50% No-switch) (Figure 1 B), similar to adults in gambling tasks who switch their responses more frequently after loss feedback [20], thus suggesting that the “prize task” was an appropriate analog to the gambling task.

### ERP results

Grand-average ERP waveforms for the two prize stimuli (good vs. bad) are shown in Figure 2. A 2×2 two-way repeated measures ANOVA with factors of Prize (good, bad) and Laterality (left, right) revealed main effects of Prize on P1 amplitude and latency, indicating that the P1 had larger amplitude and longer latency for good prizes compared to bad prizes (Amplitude: 19.95 µV vs.15.76 µV, 95% confidence interval of the difference: 1.31–7.08 µV, *F*(1,12)  = 10.06, *p*<.01, Cohen's effect size  = 0.88; Latency: 150 ms vs. 140 ms, 95% confidence interval of the difference: 2.85–16.95 ms, *F*(1,12)  = 9.35, *p* = .01, Cohen's effect size  = 0.85). The main effect of laterality and the interaction effect between Prize and Laterality did not reach significance for either the P1 amplitude or latency.

Inspection of the grand-average waveforms suggested that both good and bad prizes elicited a negativity (FRN) with peak latency around 370 ms after prize presentation. The repeated measures ANOVA on mean amplitude of FRN with factors of Prize (good, bad) revealed that there were no significant differences between good and bad prizes.

For the PSW amplitude, a 2×2 two-way repeated measures ANOVA with factors of Prize (good, bad) and Laterality (left, right) revealed a significant interaction effect between Prize and Laterality (*F*(1, 12)  = 7.26, *p* = .02). Pairwise comparisons showed that the PSW was larger for good prizes than bad prizes in the right central parietal area (11.50 µV vs.7.03 µV, 95% confidence interval of the difference: 0.81–8.12 µV, *p* = .02, Cohen's effect size = 0.74), but this difference was not observed in the left (*p* = 0.44). The topographic map also showed right hemispheric differences in scalp electrical activity evoked by good vs. bad prizes (Figure 2). These results indicated a possible laterality effect for the PSW, evoked by stimuli with different valences.

## Discussion

The present study examined electrical brain responses to positive and negative feedback in children aged 4–5 years. We found differences between bad and good prizes (i.e., negative and positive feedback) in the P1 and a long latency component, PSW. However, despite careful attempts to make the prizes' relative valences salient and meaningful to the children, we did not observe FRN differences between bad and good prizes.

The FRN peak latency we observed was around 370 ms after prize presentation. This latency is longer than the FRN typically evoked for adults (∼270 ms) and older children (∼300 ms at 8–12 years of age), but it is generally consistent with latency shifts in ERP components for studies with younger children [21]. However, previous ERP studies in adults and two studies with older children [7], [15] observed the typical differences in FRN for positive and negative feedback, but this was not observed in our study. Although this could be due to the lack of a strong difference in the valence of outcomes for these children, it is also possible that maturational effects were responsible for this. The leading theory of the FRN states that the FRN is generated when a negative reinforcement-learning signal is conveyed to the ACC via the mesencephalic dopamine system [6]. Non-human primate studies have shown that dopaminergic innervation of the PFC increases into early adulthood, suggesting that the neural system that may produce the FRN is not fully mature until young adulthood [9], [10]. Most recently, Kelly et al. [11] used a measure of functional connectivity in a neuroimaging study of ACC maturation from late childhood to early adulthood. They found that children exhibited more diffuse patterns of local functional connectivity and fewer long-range connections, relative to adults, suggesting maturation of functional connectivity in the ACC. Thus it may not be surprising that we could not observe a valence difference in FRN in preschool-aged children. Although the ACC underlying the FRN may not mature at such a young age, children are nonetheless able to use response strategies in the prize-guessing game similar to those used by adults in gambling tasks [20].

An alternative explanation for this lack of an FRN difference between the good and bad prize may have to do with the “salience” or “value” of the prizes to the children. In the adult literature, some studies have revealed that the modulation of reward magnitude influences the FRN [22], [23]. In the present study, the children were told that each correct or incorrect guess increased the number of red or black stars, respectively, which meant that positive and negative feedback could have had the same reward magnitude. However, the feedback stimulus presented on the screen was a picture of the actual prize and thus, by extension, it may be that positive feedback might be of greater affective intensity or of higher “value” for preschoolers than negative feedback simply because the good prizes were more appealing to the children. Inspection of the waveform suggests that the negativity in the time window generally associated with the FRN in children was in fact large in both the good and bad prize conditions, indicating that the brain areas underlying this component had similar responses to the positive feedback as they did to the negative feedback in our sample of preschool-aged children. Given that other adult studies have argued that the FRN is only sensitive to the reward valence, and insensitive to the reward magnitude [5], [24], [25], [26], it will be interesting to examine how reward valence and magnitude may be differentially processed during development.

Although most studies have focused on the FRN as a reflection of reward processing, a more recent perspective is that the medial frontal cortex predicts the outcomes of an action and signals when a discrepancy is detected between the predicted and actual outcomes. According to the Predicted Response Outcome (PRO) model [27], the FRN is not a reflection of the valence or magnitude of the reward, but is rather an indication that an unexpected outcome occurred. If this theory is correct, our finding would then indicate that preschoolers failed to generate a prediction for positive feedback stimuli. In other words, preschoolers (unlike older children and adults) failed to generate a strong expectancy on the basis of their responses, rendering positive and negative outcomes equally unexpected.

Previous studies have also found that motivation can modulate the FRN [28], [29]. In the present study, the pictures of the prizes themselves served as more direct feedback stimuli than the abstract symbols (e.g., green vs. red [4]), words (“correct” vs. “incorrect” [7]), or number values [5], that were used as positive and negative feedback to indicate the gain and loss of money in previous studies, and this may have increased the motivation of children to perform the task. Therefore, in the present study, we cannot exclude the possibility that the lack of difference between positive and negative feedback might be due to the paradigm and stimuli we used.

Finally, one additional interpretation that must be considered is that the negativity that we observed for both the good and bad prizes is not, in fact, the FRN, but is another fronto-centrally distributed negative component, the N300. The N300 has been reported in previous studies using pictorial stimuli and is thought to reflect early categorization processes for visually presented objects [30], [31], [32], [33]. Thus, in the present study, it may be that an FRN was not elicited at all in these preschool-aged children, but instead both sets of prizes elicited simple categorization responses at this early stage of processing followed by a later evaluative component which was reflected in the PSW. Nonetheless, in the present study we have only one age group of children. This is therefore an important and interesting finding for follow-up with multiple age groups and/or additional tasks to further clarify the nature of the FRN effects found in the present study.

Unlike the FRN, we observed that the PSW was larger for good prizes than bad prizes at the right central parietal scalp sites. Groen et al. [16] reported a similar late positivity in 10–12-year-olds, which was larger for negative than positive feedback. In many adult studies of affective pictures, a PSW in the 500–900 ms window has been found to be larger for affectively valenced (pleasant/unpleasant) than neutral pictures, suggesting it is related to emotional arousal [34]. More recently, Hajcak and Dennis [35] in their study of 5- to 8-year-old children also reported late positive potentials at occipital-parietal recording sites which increased following pleasant/unpleasant pictures compared to neutral pictures. Cuthbert et al. [36] further found that the PSW was enhanced for pictures that were more emotionally intense. In our study, the PSW was larger for good prizes than bad prizes, suggesting children may have experienced stronger emotions when the feedback was a good prize than the negative emotions they experienced when the feedback was a bad prize, since either way they are “gaining” rather than “losing” something even though they would not have been expected to like the bad prizes (Cole, 1986).

In addition, the PSW showed scalp asymmetries in our study, and this is in line with some adult studies with affectively valenced pictures [37]. These results would lead further support to the right-hemisphere hypothesis which proposes that the right hemisphere is specialized for the perception, expression, and experience of emotion [38]. However, further research with imaging technologies that afford more precise source localization capabilities such as functional Near-Infrared Spectroscopy (fNIRS) or fMRI would need to confirm the evidence for this hypothesis from a developmental point of view.

Finally, we found that the P1 amplitude over the occipital sites was larger and latency shorter for good prizes than bad prizes. The P1 is sensitive to physical stimulus parameters and reflects early visual processing and attentional manipulations [39]. We may have inadvertently caused this if the good prizes in our study were more attention-grabbing or perceptually salient, compared to bad prizes. In fact, the good prizes that the children usually selected (e.g., tambourines) were more colorful and visually complex than the bad prizes (e.g., a black bottle cap).

However, it is also possible that the P1 observed in our study is more relevant to affective responses elicited by the receipt of a “bad” vs. a “good” prize. In studies with affective pictures, the P1 has been found to be related to affective valence [34]. It thus may also be argued that the P1 in the present study might reflect early emotional processing of stimulus valence, since negative and positive emotions may be elicited when children see the feedback from having guessed a “bad” vs. a “good” prize, respectively. This interpretation is consistent with two recent studies in which pleasant pictures evoked a larger P1, compared to unpleasant or neutral pictures [40], [41]. However, other studies reported a larger P1 for unpleasant pictures than pleasant and neutral pictures [34]. These findings suggest that the P1 valence effect might be evoked by increased attention to salient image content, such as a threat (e.g., a spider) in most studies of affective pictures. In our study, the “good” prizes might have similarly evoked such a P1 response simply because of their bright and attractive colors. Nonetheless, as with the PSW findings, future studies will need to control both visual complexity and affective valence to disentangle these hypotheses.

Overall, our study suggests that preschoolers' brains appear to be more responsive to positive feedback than to negative feedback, as reflected in increased brain electrical activity (i.e., greater P1 and PSW) to positive feedback. Positive feedback has been found to increase intrinsic motivation [42], [43]. The intense focus on positive feedback that is often present in the preschool and early school years might enhance motivation for learning, which has adaptive significance for both cognitive and emotional development during this period [44], [45]. Our results are also consistent with a right-hemispheric dominance of emotion processing in young children [46], [47]. In addition, we created a child-friendly task appropriate for neuroimaging research in which a discrete and briefly experienced positive or negative emotion can be induced. Tasks have been designed to examine the neural basis of specific aspects of emotional processing such as viewing pictures of angry or happy faces [48]. However, these studies are more focused on children's *recognition* of emotional expressions. The task we designed in the present study allows us to investigate children's emotional *experiences* and their subsequent brain and behavioral regulation of these experiences. Further research on how the brain matures in its processing of emotional experiences is clearly needed and a clear limitation of the present study is that we had only one age group of children performing our task. Nonetheless, the present study offers both a method and a window of understanding for how preschoolers experience emotions and how their brains respond to both positive and negative feedback.
